# AIMP3 inhibits cell growth and metastasis of lung adenocarcinoma through activating a miR‐96‐5p‐AIMP3‐p53 axis

**DOI:** 10.1111/jcmm.16344

**Published:** 2021-02-04

**Authors:** Liting Ding, Yang Fang, Yong Li, Qinghua Hu, Meiling Ai, Keyu Deng, Xuan Huang, Hongbo Xin

**Affiliations:** ^1^ The National Engineering Research Center for Bioengineering Drugs and the Technologies the Institute of Translational Medicine Nanchang University Nanchang China; ^2^ Department of Anesthesiology the First Affiliated Hospital of Nanchang University Nanchang China

**Keywords:** AIMP3, cell growth, metastasis, miR‐96‐5p, non‐small cell lung cancer, p53

## Abstract

Aminoacyl‐tRNA synthetase‐interacting multifunctional protein‐3 (AIMP3) is a tumour suppressor, however, the roles of AIMP3 in non‐small cell lung cancer (NSCLC) are not explored yet. Here, we reported that AIMP3 significantly inhibited the cell growth and metastasis of NSCLC (lung adenocarcinoma) in vitro and in vivo. We have firstly identified that AIMP3 was down‐regulated in human NSCLC tissues compared with adjacent normal lung tissues using immunohistochemistry and western blot assays. Overexpression of AIMP3 markedly suppressed the proliferation and migration of cancer cells in a p53‐dependent manner. Furthermore, we observed that AIMP3 significantly suppressed tumour growth and metastasis of A549 cells in xenograft nude mice. Mechanically, we identified that AIMP3 was a direct target of miR‐96‐5p, and we also observed that there was a negative correlation between AIMP3 and miR‐96‐5p expression in paired NSCLC clinic samples. Ectopic miR‐96‐5p expression promoted the proliferation and migration of cancer cells in vitro and tumour growth and metastasis in vivo which partially depended on AIMP3. Taken together, our results demonstrated that the axis of miR‐96‐5p‐AIMP3‐p53 played an important role in lung adenocarcinoma, which may provide a new strategy for the diagnosis and treatment of NSCLC.

## 1 INTRODUCTION

Lung cancer, one of the most common cancers in Europe^1^ and the United States,^2^ is now the leading cause of deaths from malignant tumours in China.^3^ It is associated with a late phase (a distant stage) at initial diagnosis, high mortality, low cure rates and common long‐term side effects of treatment.^4^, ^5^ Lung cancers can be divided into two categories based on the histology of the tumours: small cell lung carcinoma (SCLC) and non‐small cell lung carcinoma (NSCLC).^2^ NSCLC constitutes over 80% of all lung cancers and can be divided into three subtypes: adenocarcinoma, squamous cell and large cell carcinoma. Although the disease could be controlled with classical doublet chemotherapy in advanced, metastatic non‐small cell lung cancer is usually restricted to only a few months^6^ and the 5‐year survival rate is terrifically less than 5%.^7^ Therefore, an optimized treatment outcome for NSCLC is eager to find novel therapeutic agents.

Aminoacyl‐tRNA synthetase‐interacting multifunctional protein‐3 (AIMP3)/p18 is an ancillary component of the macromolecular synthetase complex that initiates mammalian translation.^8^ Besides, it was reported that AIMP3 took part in diverse biological processes, such as DNA damage responding, aeging, tumorigenesis and early embryonic development. AIMP3 deletion leads to accumulation of DNA damage and loss of stemness in mouse embryonic stem cells^9^ and induces acute radiation syndrome‐like phenotype in mice,^10^ indicating that AIMP3 is involved in genome stability regulation. Kim *et al* reported that AIMP3 enhanced mitochondrial respiration and suppressed autophagic activity in stem cells.^11^ In addition, AIMP3 played important roles in p53‐mediated tumour‐suppressive response to oncogenic stresses and DNA damage through differential activation of ATM and ATR in cancer cells.^12^, ^13^ Also, a reduced AIMP3 expression has been observed in some other cancers including bladder cancer,^14^ gastric and colorectal cancer,^15^ etc. However, the roles of AIMP3 in NSCLC have not been explored in detail yet.

MicroRNAs (miRNAs) are 18‐24 nt endogenous non‐coding RNAs that negatively regulate gene expression by binding to the 3′‐untranslated region of corresponding target messenger RNAs.^16^ In human carcinoma tissues, miRNA‐induced regulation has a pivotal role in maintaining a biological process of proliferation, differentiation and apoptosis.^17^ MiRNA‐96 is one member of the miR‐183 gene family. MiR‐96 was highly expressed in many clinic tumour tissue samples, which was found to serve as an important regulator in biological behaviour of cancer cells, including prostate cancer,^18^ breast cancer,^19^ pancreatic cancer,^20^ lung cancer,^21^ head and neck squamous cell carcinoma,^22^ etc. Although miR‐96 has been reported to be elevated in NSCLC,^23^ the detailed regulatory mechanism of miR‐96 in NSCLC is not fully understood.

Here, we first examined the expression of AIMP3 in clinical tissue samples and cancer cells, and then investigated the impact of AIMP3 on NSCLC both in vitro and in vivo. Also, p53 was found to be indispensable for the function of AIMP3 on NSCLC. Moreover, miR‐96‐5p was proved to directly target AIMP3 and inhibit its expression in both clinical samples and cell lines. Our results demonstrated that AIMP3 suppressed the growth and metastasis of NSCLC via p53 and under the modulation of miR‐96‐5p.

## 2 MATERIALS AND METHODS

### 2.1 Cell lines

Human non‐small cell lung cancer cell lines (H1299, A549) were purchased from American Type Culture Collection (ATCC), SPC‐A1, Calu3, SK‐MES‐1, H292 cells were gift from Dr Chao Shen and Dr Congyi Zheng (College of Life Sciences, Wuhan University). Cells were grown in RPMI 1640 medium (Gibco) supplemented with 10% FBS (Gibco) and 100 units/mL penicillin and streptomycin under an atmosphere of 5% CO_2_ at 37°C.

### 2.2 Tissue samples

Tissue specimens were collected from 43 patients with non‐small cell lung cancer who underwent surgery at the First Affiliated Hospital of Nanchang University between 2012 and 2014. All cases of NSCLC and adjacent non‐tumour tissues were diagnosed clinically and pathologically. Fast frozen tissue for protein/RNA extraction and paraffin‐embedded tissue for continued histological observation was collected. The use of human tissues was approved by the Ethics Committee of the First Affiliated Hospital of Nanchang University and conforms to the Helsinki Declaration and to local legislation.

### 2.3 Immunohistochemistry

Immunohistochemistry analyses were performed as described previously.^24^ AIMP3 staining was scored by two independent pathologists, blinded to the clinical characteristics of the patients. The scoring system was based on the staining intensity and extent. Staining intensity was classified as 0 (negative), 1 (weak), 2 (moderate) and 3 (strong). Staining extent was dependent on the percentage of positive cells (examined in 200 cells) were divided into 0 (<5%), 1 (5%‐25%), 2 (26%‐50%), 3 (51%‐75%) and 4 (>75%). The final score was obtained by multiplying the two scores and ranged from 0 to 12.

### 2.4 Vectors, RNA interference and transfection

An AIMP3 expression construct was generated by cloning full‐length human AIMP3 cDNA into the pCMV‐HA plasmid. Small interfering RNA (siRNAs) was synthesized by RiBo Bio Co. The AIMP3 siRNA sequences were siRNA1: GCAACAUCUGUCUAGUGUU; siRNA2: ACCUGACAGUUCAAGAAAA; siRNA3: CACACAGAGGUAGGAACU. Transfection of siRNA or plasmids was performed using the Lipofectamine 3000 reagent (Invitrogen) according to the manufacturer's instruction.

### 2.5 Low serum assay and saturation density assay

For low serum assay, cells were plated at a density of 10^5^ cells in 12‐well plates and allowed to adhere overnight in 10% FBS medium. On the following day, the cell number was counted as the data of day 0, the medium was changed to 1% FBS RPMI 1640 and then changed every other day for 6 days. At the indicated times, the cells were trypsinized and counted with a hemocytometer. For saturation density assay, 10^5^ cells were seeded in 12‐well plates in 10% FBS RPMI 1640. The medium was changed every other day. The cell density was determined by counting the cells on the sixth day.

### 2.6 Colony formation assay

Five hundred cells were seeded in 6‐well dishes, and allowed to adhere overnight. On the following day, the media was changed to 5% FBS RPMI 1640. All cells were then grown for 2 weeks, with medium changed every other day. Plates were fixed with 4% formaldehyde and stained with 2% crystal violet.

### 2.7 Transwell migration assay

Transwell migration assay was performed using 8 μm pore size transwell chambers (BD Falcon). A total of 10^5^ cells in 0.2 mL medium supplemented with 1% FBS were plated in the upper chamber. The lower chamber of the transwell device was filled with 500 μL RPMI 1640 supplemented with 10% FBS. After incubation at 37°C for 10 hours, cells remaining on the upper surface of the membrane were removed. The cells on the lower surface of the membrane were fixed, stained with crystal violet and photographed under a light microscope (Olympus IX71). Of 0.2 mL DMSO was added to the upper chamber to dissolve the crystal violet, and then the absorbance of the samples was measured at 450 nm using a Multiskan FC microplate reader (Thermo Fisher Scientific).

### 2.8 Western blot and immunoprecipitation (IP)

The proteins from tissues and cells were separated by standard 10% SDS‐PAGE followed by transferring the proteins to a PVDF membrane. The proteins were detected by the following primary antibodies: AIMP3 (Proteintech, 10805‐1‐AP), p53 (Proteintech, 10442‐1‐AP), N‐cadherin(Cell Signaling, 13116), E‐cadherin (Cell Signaling, 3195), Vimentin (Cell Signaling, 5741), Snail (Cell Signaling, 3879), Slug (Cell Signaling, 9585), ATM (Proteintech, 27156‐1‐AP), p‐ATM (Abcam, ab81292), β‐actin (Cell Signaling, 3700) and GAPDH (Proteintech, 60004‐1‐Ig) and followed by incubation with a secondary antibody. Staining was performed with ECL western blot detection reagent. Antibody to GAPDH or β‐actin was served as the endogenous control. All experiments were performed in triplicate.

For immunoprecipitation, cells were collected with lysis buffer and subjected to IP with HA antibodies and protein‐G beads at 4°C overnight with gentle rolling. Bound proteins were analysed by immunoblotting.

### 2.9 Quantitative real‐time RT‐PCR (qPCR)

Total RNA was extracted from tissues and cells using TRIzol^®^ Reagent (Life Technologies) according to the instructions. For each sample, 1 μg RNA was reverse‐transcribed (RT) to cDNA using PrimeScript^™^ RT reagent Kit with gDNA Eraser (TAKARA). Quantitative PCR (qPCR) was carried out using TB Green^®^ *Premix Ex Taq* ^™^ II (TAKARA) on ABI ViiA‐7 real‐time PCR system instrument. The results were normalized to GAPDH transcript or U6. All data were expressed in terms of fold‐change relative to the control samples. The primers were validated for their amplification efficiency and specificity. Sequences of the primers are listed in Table S1.

### 2.10 Luciferase assay

Cells (1 × 10^4^) were seeded in triplicate in 48‐well plates and allowed to settle for 24 hours. One hundred nanograms of luciferase reporter plasmids or the control plasmid, plus 1 ng of pRL‐TK renilla plasmid (Promega), were transfected into cells using the Lipofectamine 3000 reagent (Invitrogen). Luciferase and renilla signals were measured using the Dual Luciferase Reporter Assay Kit (Promega) according to a protocol provided by the manufacturer.

### 2.11 Cell cycle analysis and cell apoptosis assay

After 48 hours transfection of AIMP3, cells were harvested and suspended with 0.5 mL ice‐cold PBS. Then the cells were fixed in 70% alcohol on ice for over 2 hours. After being washed with PBS, cell pellet was resuspended in 1 mL of PBS solution containing 50 μg/mL RNase A and 10 μg/mL propidium iodide (PI). The cells were then incubated for 30 minutes at room temperature and analysed using the Beckman flow cytometer CytoFLEX (Beckman Coulter).Apoptosis was measured using the Annexin‐V, FITC Detection Kit (DojinDo). All the cells were collected and incubated with Annexin V/FITC and PI at room temperature for 15 minutes in the dark. Cell apoptosis was assessed by flow cytometry (Beckman Coulter). Experiments were performed in triplicate.

### 2.12 Tumour xenograft model

The mice were maintained under controlled temperature (22‐24°C) and were housed in barrier facilities with a 12 hours light‐dark cycle with free access of water and food. All mice were treated in accordance with the Guide for the Care and Use of Laboratory Animals, and approved by the Ethics Committee of Nanchang University. The mice used in this study were Balb/c‐nu/nu male nude mice (5‐6 weeks old). To evaluate the role of AIMP3 in a human lung cancer mouse model, A549 stable cell lines were subcutaneously injected into nude mice (1 × 10^7^ cells in 50 μL PBS/mouse). Tumours were measured with a caliper every 3 days, and the tumour volume was calculated using the following formula: V = (length × width^2^)/2. At day 30 after tumour cell inoculation, all experimental mice were sacrificed and the xenograft tumours were harvested. The total weight of the tumours in each mouse was measured.

### 2.13 In vivo tumour metastasis assay

Five week‐old Balb/c‐nu/nu male nude mice were injected with A549/Mock and A549/AIMP3 cells (n = 8 per group) via the tail vein (1 × 10^6^ cells in 50 μL PBS/mouse). After 60 days, mice were sacrificed by administration of an overdose of anesthetic and lungs were harvested. Visible lung surface metastatic white spots (tumour nodules) in each mouse were counted.

### 2.14 Statistical analysis

For each experiment, three independent replicates were performed. All the data were expressed as mean ± SEM The Student's *t* test was used to analyse the difference between two groups. The correlation between AIMP3 and miR‐96‐5p expression was analysed using the Pearson correlation analysis. A *P*‐value of less than .05 was considered statistically significant.

## 3 RESULTS

### 3.1 The expression of AIMP3 was down‐regulated in cancer tissues of human non‐small cell lung cancer (NSCLC)

**FIGURE 1:**
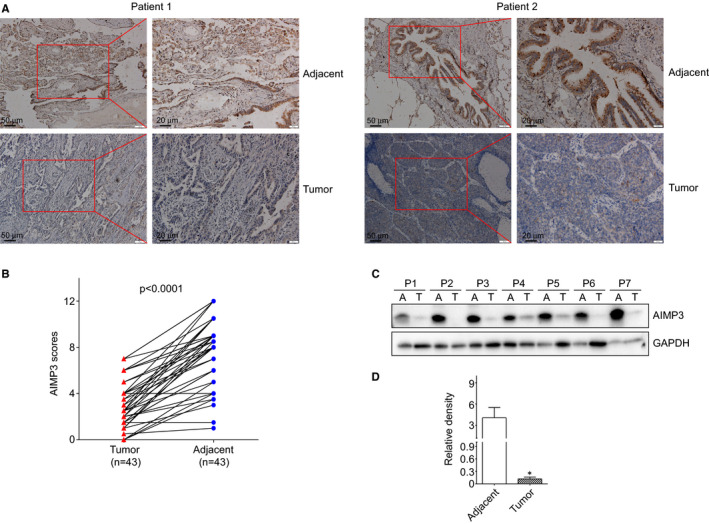
AIMP3 expression in clinical non‐small cell lung tumour and adjacent tissues. A, Representative IHC staining of AIMP3 on NSCLC and adjacent tissues. B, AIMP3 expression plotted based on immunohistochemical score. C, Seven random selected human NSCLC tumours and their paired adjacent normal tissues were tested using AIMP3 specific antibody. A, adjacent normal tissues; T, tumours. D, Density analysis of Western blot results. * *P* < .05 compared with adjacent normal tissues

To study the biological functions of AIMP3 in non‐small cell lung cancer (NSCLC), immunohistochemistry (IHC) staining of AIMP3 was performed in human NSCLC specimens. As shown in Figure 1A, the expressions of AIMP3 in tumour tissues were much lower than that in adjacent normal lung tissues. The scores of AIMP3‐positive rates in lung cancers were significantly smaller than that in normal tissues (*P* < .001) (Figure 1B). The similar results for AIMP3 protein expressions were also observed in the randomly selected 7 paired lung cancer and adjacent normal tissues measured by Western blot analysis (Figure 1C,D).

### 3.2 Overexpression of AIMP3 suppressed the proliferation and the colony formation of NSCLC cells in vitro

**FIGURE 2:**
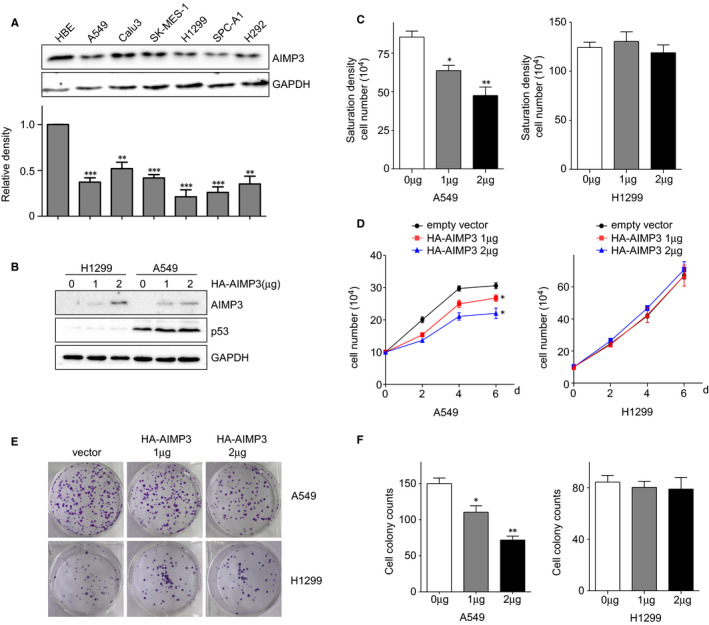
Overexpression of AIMP3 suppresses the proliferation of NSCLC cells. A, The expression of AIMP3 protein in six NSCLC cell lines indicated and HBE cells. Relative fold changes were determined by densitometry and normalized to GAPDH. B, H1299 and A549 cells were transiently transfected with different amounts of HA‐AIMP3 for 24 h. Cell lysates were extracted and used to detect the protein levels of AIMP3 and p53. C, Saturation density assay. Cells transfected with indicated amount of AIMP3 were cultured in full medium for 6 days, trypsinized and counted. Data are presented as mean ± SEM (n = 3). D, Low serum assay. Cells transiently transfected with AIMP3 were cultured in 1% FBS RPMI1640 medium. At the indicated times, cells were trypsinized and counted. Data are presented as mean ± SEM (n = 3). E, Colony formation assay. 500 transiently transfected cells were seeded in 6‐well plates in 5% FBS RPMI1640 medium, respectively. After 14 days, cells were fixed and stained with 2% crystal violet. F, The number of colonies was counted and data are presented as mean ± SEM (n = 3). *P < .05, **P < .01, ***P < .001 by Student's *t* test

Basing on the clinical results, we further investigated the effect of AIMP3 on lung cancer cell proliferation in vitro. We first examined the expression levels of AIMP3 protein in six lung cancer cell lines, A549, Calu3, SK‐MES‐1, H1299, SPC‐A‐1 and H292. As showed in Figure 2A, the expressions of AIMP3 were significantly reduced in all of the lung cancer cell lines compared with normal lung tissue cell lines (HBE, bronchial epithelial cells), which was consistent with the results of clinical samples. It has been reported that AIMP3 was a haploinsufficient tumour suppressor with activation of p53,^12^ and our results also showed that the mRNA expressions of both AIMP3 and p53 in lung cancer cell lines were markedly attenuated compared with normal lung tissues cell line (Figure S1). To explore whether the up‐regulation of AIMP3 suppresses NSCLC progression, p53‐positive A549 and p53‐negative H1299 cells were transfected with different amounts of HA‐AIMP3, separately (Figure 2B). The growth and colony formation of the transfected cancer cells were detected after 24 hours of transfection. As shown in Figure 2C,D, overexpression of AIMP3 significantly inhibited the growth of A549 cells both in low serum medium and full growth medium (saturation density), but didn't affect the growth of H1299 cells. As expected, the up‐regulation of AIMP3 significantly decreased the colony formation of A549 cells but not H1299 cells (Figure 2E,F). Taken together, overexpression of AIMP3 dramatically inhibits the proliferation of A549 cells but not H1299 cells, indicating that the inhibition effect of AIMP3 on lung cells was dependent on the activation of p53. Accordingly, knockdown of AIMP3 promoted the proliferation of A549 cells but not H1299 cells (Figure S2). These results suggested that knockdown of AIMP3 promoted lung cancer cell proliferation in a p53‐dependent manner in vitro.

### 3.3 AIMP3 suppressed the migration of NSCLC cells in vitro

**FIGURE 3:**
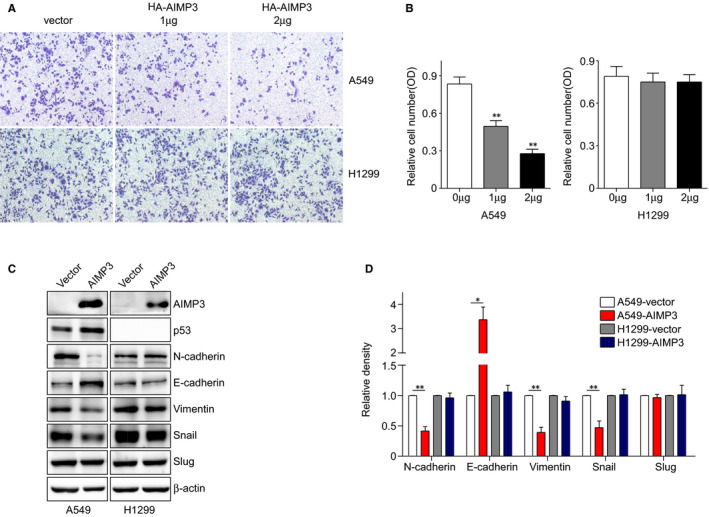
AIMP3 suppresses the migration of NSCLC cells. A, Transwell migration assay. 10^4^ transiently transfected H1299 and A549 cells were cultured in 1% FBS RPMI1640 medium and seeded in transwell chambers. 10 h later, cells were fixed, stained and photographed. B, The crystal violet was dissolved in DMSO, and the absorbance was tested. Data are presented as mean ± SEM (n = 3). C, Cells were transiently transfected with AIMP3 or control vector. The expression of N‐cadherin, E‐cadherin, Vimentin, Snail and Slug was analysed by Western blot. D, Relative fold changes were determined by densitometry and normalized to β‐actin

We further examined the effect of AIMP3 on migration of NSCLC cells via transwell assays. As shown in Figure 3A,B, the numbers of migrated A549 cells were decreased when AIMP3 was over‐expressed, but not H1299 cells. Furthermore, we evaluated the effects of AIMP3 on the expression of several EMT‐related markers. Western blot and the related Gel‐analysis results showed that up‐regulation of AIMP3 suppressed the expression of Vimentin and N‐cadherin, but increased the expression of E‐cadherin protein only in A549 cells. Also, the expression of EMT‐related transcription factor Snail was significantly decreased, whereas expression of Slug had no obvious change (Figure 3C,D). In order to confirm the effects of AIMP3 on NSCLC cells, we down‐regulated AIMP3 using siRNAs in both A549 and H1299 cells and then performed the transwell assays above. Down‐regulation of AIMP3 in A549 cell lines dramatically increased the cell migration (Figure S3) but had no effect on the migration of H1299 cells.

### 3.4 AIMP3‐induced cell cycle arrest and apoptosis was dependent on the activation of p53

**FIGURE 4:**
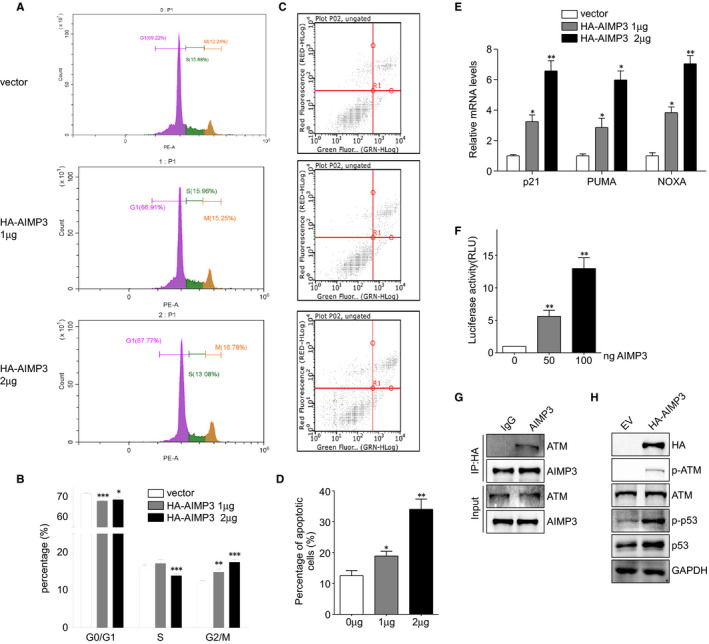
AIMP3 induces cell cycle arrest at G2/M phase and apoptosis depends on p53. A, Cell cycle analysis of A549 cells transfected with different amounts of AIMP3 for 48 h. Cells were collected and analysed by flow cytometry after propidium iodide staining. The inserts showed the proportion of cells for each phase and were marked with different colours (violet: G0/G1 phase, green: S phase and gold: G2/M phase). B, The quantification of cell number in each phase of cell cycle. C, Examination of apoptotic cells in A549 cells transfected with indicated amount of AIMP3. D, Quantitative analysis of the percentage of apoptotic cells was shown in Figure C. Data are presented as mean ± SEM (n = 3); * *P* < .05, ** *P* < .01. E, The effect of AIMP3 on p53‐dependent transcription of *NOXA*, *PUMA* and *p21*. A549 cells were transiently transfected with indicated amount of AIMP3 and incubated for 24 h. Total RNAs were extracted and used for RT‐PCR detection. F, A549 cells were co‐transfected with p21 promoter‐reporter vector and different amounts of AIMP3 expression plasmid (0, 50, 100 ng). Relative luciferase activity values are presented as mean ± SEM (n = 3); ** *P* < .01 by Student's t test. G, A549 cells were transiently transfected with HA‐AIMP3 for 24 h, and whole cell lysates were immunoprecipitated with HA antibody (mouse) or mouse IgG as control. The precipitates were immunoblotted with ATM antibody. H, Cells were transiently transfected with AIMP3 or control vector. The expression of indicated proteins was analysed by Western blot

To further investigate the role of AIMP3 in lung cancer cell proliferation, cell cycle was analysed in A549 cells by employing flow cytometry after propidium iodide staining. The results showed that up‐regulation of AIMP3 increased the proportion of G2/M cells (Figure 4A,B). Then, Annexin V‐FITC/PI staining assay was performed to evaluate the role of AIMP3 in cell apoptosis in A549 cells. As shown in Figure 4C,D, overexpression of AIMP3 induced the cell apoptosis in a dose‐dependent manner. Next, we determined whether up‐regulation of AIMP3 would enhance the p53‐dependent transcription via checking the mRNA levels of p53‐targeted genes *NOXA*, *p21* and *PUMA*. The results showed that the expressions of all three genes tested were enhanced by AIMP3 in a dose‐dependent manner (Figure 4E). The effect of AIMP3 on p21 transcription was also checked by a luciferase assay. Consistently, the relative luciferase activities of p21 promoter were elevated when AIMP3 was overexpressed (Figure 4F). Park et al reported that AIMP3 promoted p53 expression via interactions with ATM in HCT116 cells.^12^ To clarify how AIMP3 regulated p53 in lung cancer cells, we tested whether AIMP3 interacts with ATM by co‐immunoprecipitation. Results of Figure 4G showed that AIMP3 can interact with ATM. Moreover, ectopic expression of AIMP3 increased the phosphorylation of ATM and p53 (Figure 4H). These results indicated that AIMP3 up‐regulated p53 through binding to ATM.

### 3.5 AIMP3 inhibits tumour growth and metastasis in vivo

**FIGURE 5:**
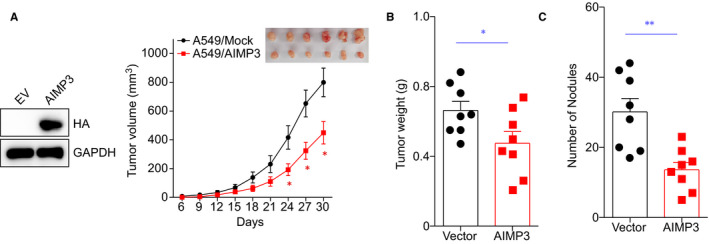
AIMP3 suppresses tumour growth in mouse xenograft model. A, A549 cells stably expressing empty vector or AIMP3 were subcutaneously injected into nude mice, and the expression of AIMP3 was confirmed by western blot. Tumour volume was measured every 3 days and is shown as the mean ± SEM (empty vector, Mock: n = 8, AIMP3: n = 8). Significant differences were determined by one‐way ANOVA, followed by Dunnett's test; * *P* < .05 (versus empty). A representative image of tumour xenografts harvested at day 30. B, Tumour weights from the two groups are represented. C, BALB/c nude mice (n = 8 per group) received a tail vein injection of A549 cells stably expressing empty vector or AIMP3. The number of metastatic lung nodules in individual mice was counted under a dissection microscope. Data represent the mean ± SEM * *P* < .05 vs empty vector

To evaluate the effect of AIMP3 on tumour growth of NSCLC in vivo, we performed xenograft experiments in nude mice. To this end, we established stable AIMP3 expressed cell lines A549, and the expression of AIMP3 was confirmed by western blot (Figure 5A). The cells stably expressing AIMP3 or empty vector were injected subcutaneously into the right axillary region of nude mice, and the volumes of xenograft tumours were measured every 3 days. One month later, tumours were isolated and imaged. As showed in Figure 5A (right panel), we found that xenograft tumours derived from A549 cells that stably expressed AIMP3 exhibited significantly slower growth rates and smaller tumour sizes, on average, compared with cells expressing the empty vector. In addition, the average wet weight of tumours from nude mice injected with A549 cells stable expressed AIMP3 was less than that from nude mice injected with control A549 cells (Figure 5B). To further investigate the effect of AIMP3 on tumour metastasis of NSCLC in vivo, a lung metastasis model in nude mice was established. Nude mice injected with stable AIMP3 expressed A549 cells via the tail vein. The numbers of lung tumour nodules in nude mice injected with the stable AIMP3 expressed A549 cells were significantly lower than that in animals injected with the control cells after 60 days of inoculation (Figure 5C).

### 3.6 AIMP3 was a direct target of miR‐96‐5p in NSCLC cells

**FIGURE 6:**
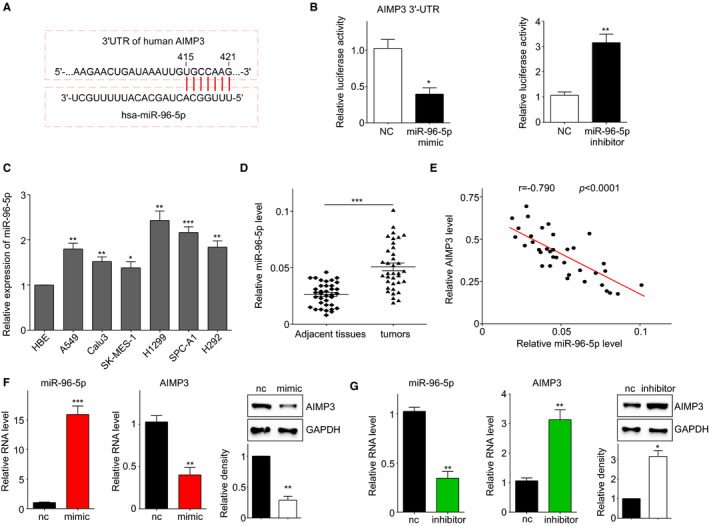
*AIMP3* is a direct target of miR‐96‐5p. A, Prediction of miR‐96‐5p binding sites in 3’UTR of *AIMP3* using Target Scan Human 7.2 software. B, A549 cells were co‐transfected with AIMP3 3’‐UTR plasmid and miR‐96‐5p mimic (left panel) or miR‐96‐5p inhibitor (right panel). Relative luciferase activity values are presented as mean ± SEM (n = 3); * *P* < .05, ** *P* < .01. C, The expression of miR‐96‐5p in six NSCLC cell lines and HBE cells. U6 was used as an internal control. * *P* < .05, ** *P* < .01, *** *P* < .001. D, miR‐96‐5p expression in 36 pairs of NSCLC and matched adjacent normal tissues. *** *P* < .001. E, Correlation between AIMP3 and miR‐96‐5p expression in 36 NSCLC tissues. F, Up‐regulation of miR‐96‐5p suppressed the expression of AIMP3 protein and mRNA in A549 cells. G, Inhibition of miR‐96‐5p increased both the mRNA and protein levels of AIMP3

The above results indicated that AIMP3 expression was down‐regulated in tumour tissues and NSCLC cells, but the regulatory mechanism remains unclear. To clarify the upstream modulator of AIMP3, Target Scan Human 7.2 software was used to predict the potential miRNA which may bind to 3’UTR of human *AIMP3* gene. We found that the expression of AIMP3 may be under control of miR‐96‐5p (Figure 6A). In Figure 6B, miR‐96‐5p mimics significantly inhibited the luciferase activity of AIMP3 3′‐UTR in A549 cells, whereas its inhibitor increased the luciferase activity, indicating that AIMP3 was a direct target of miR‐96‐5p. Next, we examined the levels of miR‐96‐5p both in cancer cell lines and paired clinic samples (tumours and matched adjacent normal tissues) using qRT‐PCR. As shown in Figure 6C, the expression level of miR‐96‐5p was elevated in all six cancer cells tested compared with bronchial epithelial cells HBE. And the miR‐96‐5p expression level was dramatically induced in NSCLC tissues, as compared with adjacent normal tissues (Figure 6D). Furthermore, we analysed the relationship between AIMP3 and miR‐96‐5p expression in 36 paired clinic samples, with the results revealed an obvious negative correlation between AIMP3 and miR‐96‐5p (Figure 6E). Moreover, miR‐96‐5p mimics suppressed both mRNA level and protein level of AIMP3 (Figure 6F), whereas its inhibitors increased AIMP3 mRNA and protein expressions (Figure 6G). Collectively, these results indicated that AIMP3 was a direct target of miR‐96‐5p.

### 3.7 miR‐96‐5p promoted the growth and migration of lung cancer cells both in vitro and in vivo

**FIGURE 7:**
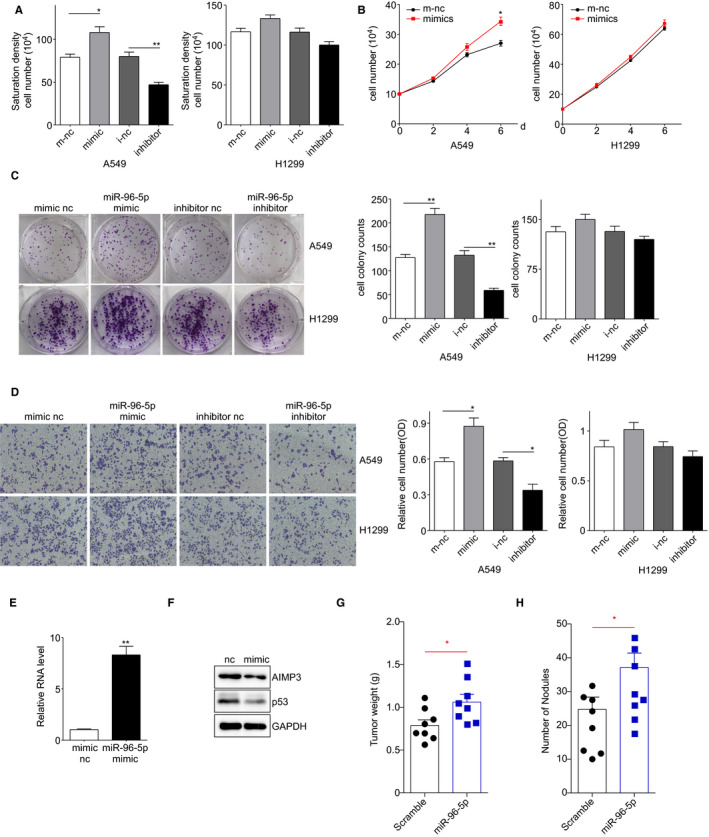
The effect of miR‐96‐5p on the growth of NSLSC cells. A, Saturation density assay. H1299 and A549 cells transiently transfected with miRNA control, miR‐96‐5p mimic or inhibitor were cultured in full medium. 6 days later, cells were trypsinized and counted. Data are presented as mean ± SEM (n = 3); * *P* < .05, ** *P* < .01 by Student's *t* test. B, Low serum assay. H1299 and A549 cells transiently transfected with miRNA control, miR‐96‐5p mimic were cultured in 1% FBS RPMI1640 medium. Cells were trypsinized and counted at indicated times. Data are presented as mean ± SEM (n = 3); ** *P* < .01 by Student's *t* test. C, Colony formation assay. Five hundred transiently transfected H1299 and A549 cells were seeded in 6‐well plates in 5% FBS RPMI1640 medium, respectively. After 14 days, cells were fixed and stained. The number of colonies was counted and data are presented as mean ± SEM (n = 3); ** *P* < .01 by Student's *t* test. D, Transwell migration assay. 10^4^ transiently transfected H1299 and A549 cells were cultured in 1% FBS RPMI1640 medium and seeded in transwell chambers. 10 h later, cells were fixed, stained and photographed. Then, the crystal violet was dissolved in DMSO and the absorbance was tested. Data are presented as mean ± SEM (n = 3); ** *P* < .01 by Student's *t* test. E, qPCR was used to examine the miR‐96‐5p level in transfected A549 cells. F, Western blot was performed to detect the expression of AIMP3 and p53 in these cells. G, Tumour weights of tumours from control or miR‐96‐5p mimic transfected A549 xenografts at sacrifice. n = 8 for each group. H, The number of metastatic lung nodules in individual mice received a tail vein injection of A549 cells transfected control or miR‐96‐5p mimic was counted. Data represent the mean ± SEM* *P* < .05 vs control

To elucidate the role of miR‐96‐5p in NSCLC cell progression and its mechanism, we transfected miR‐96‐5p mimic and inhibitor into A549 and H1299 cells, respectively. miR‐96‐5p mimic dramatically promoted the growth of A549 cells both in low serum medium and full growth medium, which had slight effects on the growth of H1299 (Figure 7A,B). As shown in Figure 7C, miR‐96‐5p significantly increased the colony formation of A549 cells but had only slight influence on H1299 cells. Based on the transwell assay results, we observed that miR‐96‐5p only promoted the migration of A549 cells but had a slight effect on H1299 cells (Figure 7D). These results suggested that miR‐96‐5p facilitated the growth and migration of lung cancer cells mainly via AIMP3 and p53.

To further confirm the correlation between miR‐96‐5p and AIMP3 in human lung cancer growth and metastasis in vivo, we performed xenograft experiments in nude mice. Firstly, AIMP3 and p53 protein levels were analysed using western blot and RT‐qPCR to validate the constructed cell lines (Figure 7E,F). Next, we used these cells to generate a mouse xenograft cell metastasis model. Approximately one month after subcutaneous implantation of A549 control and A549 miR‐96 cells, the tumour weight of the A549 miR‐96 group was significantly higher than that of the control group (Figure 7G). In addition, there were significantly more lung nodules in the A549 miR‐96 nude mice than in the A549 control nude mice (Figure 7H). Collectively, the results clearly demonstrated that the decreased AIMP3 protein level caused by miR‐96‐5p triggered xenograft tumour formation.

## 4 DISCUSSION

Aminoacyl‐tRNA synthetases (ARSs) are a family of highly conserved enzymes that link amino acids to the specific tRNAs during translation process. The nine ARSs and three nonenzymatic factors AIMPs form a macromolecular complex, which plays an important role in protein synthesis and some other biological processes.^25^ AIMP3, also known as eukaryotic translation elongation factor 1 epsilon‐1(EEF1E1) or p18, was reported as a tumour suppressor.^12^ Lower expression of AIMP3 was only mentioned in bladder cancer^14^ and gastric/colon cancer^15^ with clinical tissue samples. No study to date has reported the effects of AIMP3 on NSCLC. So, we first examined the expression of AIMP3 in clinical NSCLC tumours and adjacent normal lung tissues via IHC and western blot analysis. We observed that AIMP3 was down‐regulated in NSCLC tissues, which is consistent with the previous studies on other tumours.

AIMP3 is a haploinsufficient tumour suppressor since the heterozygous knockout mice are more susceptible to spontaneous tumours.^12^ In response to DNA damage and oncogenic stress, AIMP3 was up‐regulated and translocated into the nucleus, which directly interacted with ATM/ATR to induce the expression of p53.^12^, ^13^ In the present study, we observed that AIMP3 suppressed the proliferation and migration of A549 cells but did not have any effect on H1299 cells which is lack of p53. Overexpression of AIMP3 increased the mRNA expression of the target gene (*PUMA*, *NOXA* and *p21*) of p53 and the luciferase activities of p21. These results indicated that AIMP3 functions as a tumour suppressor in NSCLC cells dependent of p53. Since the effects of AIMP3 on tumour growth or metastasis in vivo were unclear, we explored the roles of the protein in xenograft nude mice models using stable AIMP3‐expressed cells. Our results showed that AIMP3 inhibited the tumour growth and metastasis of NSCLC cell line A549 but not H1299, indicating that AIMP3 could be regarded as a tumour suppressor in vivo only in the present of p53.

It has been proved that AIMP3 is down‐regulated in several tumours compared with adjacent normal tissues.^12^, ^14^, ^15^ However, the regulatory mechanism is seldomly explored. In order to elucidate the molecular mechanism, we searched the upstream regulator of AIMP3 using Target Scan Human 7.2 software and found that AIMP3 was a direct target of miR‐96‐5p. Compared with normal tissues, miR‐96‐5p was up‐regulated and there was a convinced negative correlation between the expression of AIMP3 and miR‐96‐5p. Recently, Cai *et al* reported that miR‐96‐5p acting as an oncogene and may play an important role in the development and progression of NSCLC, but the mechanism is still unknown.^23^ Guo *et al* observed that miR‐96 promoted the growth of NSCLC cells via down‐regulating RECK.^26^ Also, miR‐96 can target SMAD9 to suppress cisplatin‐induced apoptosis and induce cisplatin chemoresistance in NSCLC.^27^ Our results showed that miR‐96‐5p promoted the growth and migration of NSCLC cells partially depending on AIMP3. Moreover, we also observed that miR‐96‐5p could trigger xenograft tumour formation and metastasis of A549 cells for the first time.

In conclusion, we found that *AIMP3* as a novel tumour suppressor significantly inhibited the cell growth and metastasis of NSCLC in a p53‐dependent manner in vitro and in vivo. Furthermore, we identified that AIMP3 was a direct target of miR‐96‐5p, which was down‐regulated in both clinical tumour tissues and cancer cell lines. Finally, we demonstrated that the miR‐96‐5p‐AIMP3‐p53 axis played a key role in NSCLC and our findings should provide a new target and strategy for the treatment of NSCLC clinically.

## CONFLICT OF INTEREST

The authors declare that they have no conflict of interest.

## AUTHOR CONTRIBUTIONS

**Liting Ding:** Investigation (equal); Methodology (lead); Project administration (equal). **Yang Fang:** Investigation (equal); Methodology (equal); Project administration (equal); Resources (supporting). **Yong Li:** Conceptualization (supporting); Data curation (equal); Formal analysis (equal); Funding acquisition (supporting); Writing‐review & editing (supporting). **Qinghua Hu:** Investigation (supporting); Methodology (supporting); Project administration (supporting). **Meiling Ai:** Methodology (supporting); Project administration (supporting). **Ke‐Yu Deng:** Investigation (supporting); Project administration (supporting). **Xuan Huang:** Conceptualization (lead); Data curation (equal); Funding acquisition (lead); Supervision (equal); Writing‐original draft (lead). **Hong‐Bo Xin:** Conceptualization (supporting); Supervision (equal); Writing‐review & editing (lead).

## ETHICAL APPROVAL

The use of human tissues was approved by the Ethics Committee of the First Affiliated Hospital of Nanchang University and conforms to the Helsinki Declaration and to local legislation. The patients/participants provided their written informed consent to participate in this study. All the experimental procedures were approved by Nanchang University Institutional Animal Research Committee and were carried out in accordance with Jiangxi Province Laboratory Animal Care Guidelines for use of animals in research.

## Supporting information

Fig S1

Fig S2

Fig S3

Table S1

## Data Availability

All datasets generated for this study are included in the article/Supplementary Material.
