# Supplementary figures and images for: AIMP3 inhibits cell growth and metastasis of lung adenocarcinoma through activating a miR‐96‐5p‐AIMP3‐p53 axis
Source: J Cell Mol Med. 2021 Feb 4;25(6):3019–30. doi: 10.1111/jcmm.16344 (PMC7957209; doi:10.1111/jcmm.16344)

Supplementary Figure 1

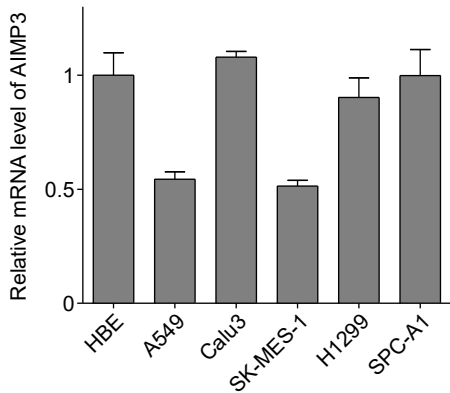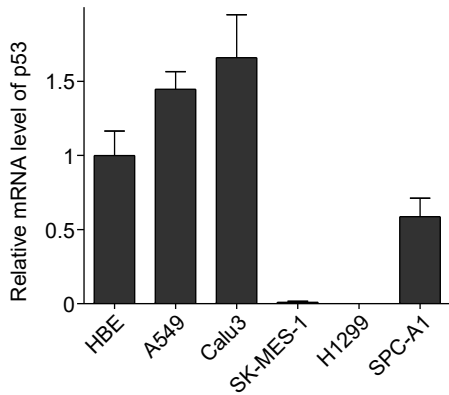

Supplement: Supplementary file 1 — Fig S1 [file JCMM-25-3019-s002.pdf]

Supplementary Figure 2

**A**

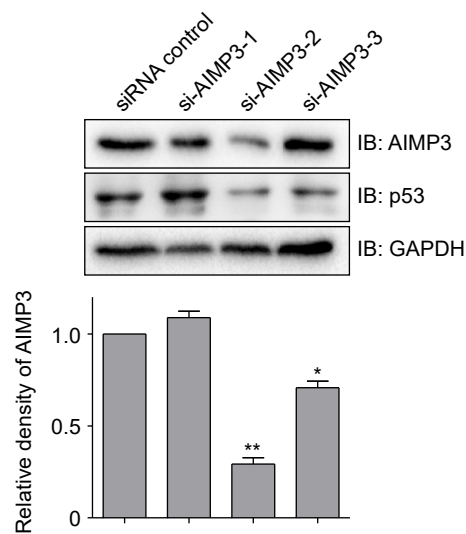

**B**

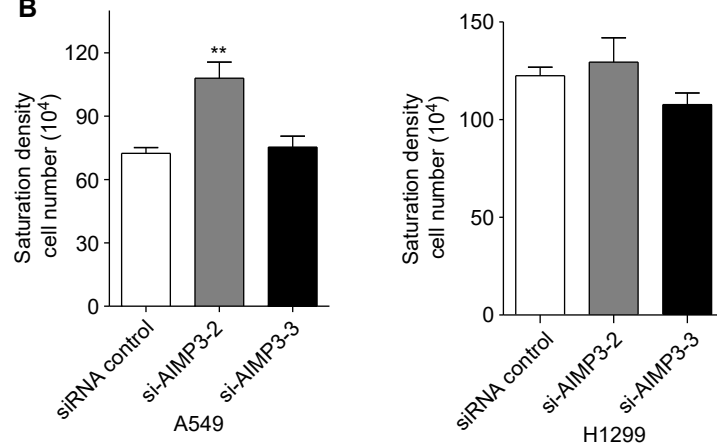

**C**

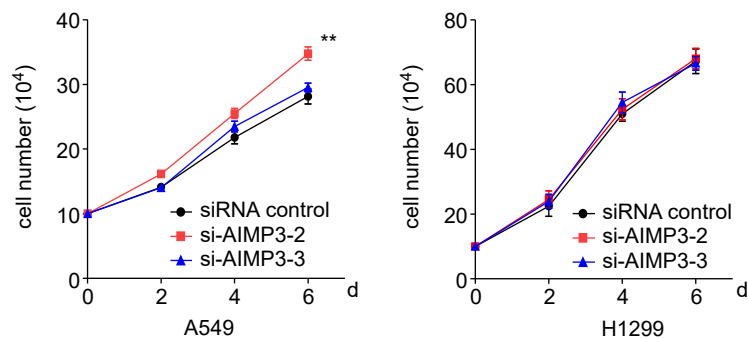

**D**

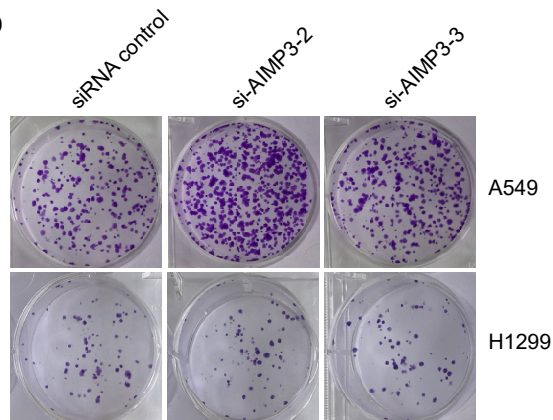

**E**

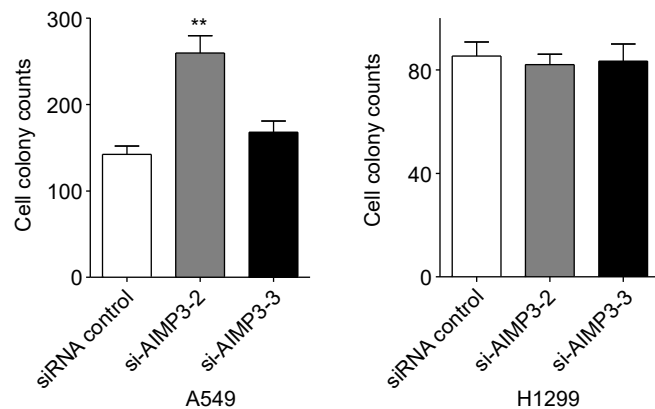

Supplement: Supplementary file 2 — Fig S2 [file JCMM-25-3019-s001.pdf]

Supplementary Figure 3

**A**

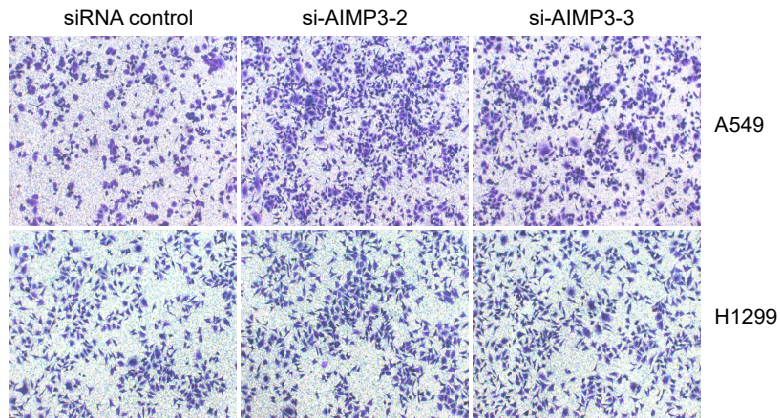

**B**

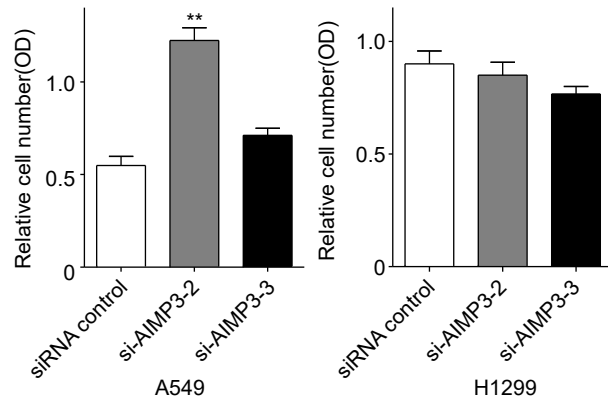

Supplement: Supplementary file 3 — Fig S3 [file JCMM-25-3019-s003.pdf]
