# Supplementary material for: AIMP3 inhibits cell growth and metastasis of lung adenocarcinoma through activating a miR‐96‐5p‐AIMP3‐p53 axis
Source: J Cell Mol Med. 2021 Feb 4;25(6):3019–30. doi: 10.1111/jcmm.16344 (PMC7957209; doi:10.1111/jcmm.16344)
Supplement: Supplementary file 4 — Table S1 [file JCMM-25-3019-s004.docx]

**Supplementary Table 1: Sequences of QPCR primers used in this study.**

| **Primer Name** | **Forward Sequences (5’-3’)** | **Reverse Sequences (5’-3’)** |
| --- | --- | --- |
| AIMP3 | TGGGACTGAGTAAGGGGAATA | TTGGCTTGCTTGACTAGATGA |
| p53 | GAGGTCGGAGCTTGTCGAG | TTCTTGGCTGAGTTTCTGCAC |
| p21 | GAACTTCGACTTTGTCACCG | TGCCTCCTCCCAACTCAT |
| PUMA | GCGGACGACCTCAACG | GGGTGCAGGCACCTAATT |
| NOXA | CAGCAGAGCTGGAAGTCGA | CCTGAGCAGAAGAGTTTGGAT |
| GAPDH | CAGGGCTGCTTTTAACTCTGGT | GATTTTGGAGGGATCTCGCT |
| miR-96-5p | TTTGGCACTAGCACAT | GAGCAGGCTGGAGAA |
| U6 | GTGCGTGTCGTGGAGTCG | AACGCTTCACGAATTTGCGT |
